# Treatment of gastrointestinal complication in transthyretin amyloidosis. A single centre’s experience

**DOI:** 10.1186/1750-1172-10-S1-I23

**Published:** 2015-11-02

**Authors:** Ole B Suhr

**Affiliations:** 1 Department of Public Health and Clinical Medicine, Umeå University, Umeå, Sweden

## Background

Gastrointestinal complications have a substantial impact on ATTR amyloidosis patients’ survival and quality of life.

The disturbances are generally attributed to motility disturbances caused by autonomic denervation, but the pathogenesis is poorly defined. The most common symptoms are those of early satiety, nausea and vomiting from the upper gastrointestinal (GI) tract, and constipation, constipation alternating with diarrhoea, or continuous diarrhoea from the lower. Faecal incontinence is common in later stages of the disease and has a devastating impact on the patient’s quality of life.

## Evaluation and treatment

Upper endoscopy and scintigraphic visualisation of gastric emptying can diagnose gastric retention often before the patients develop symptoms. For treatment, motilin agonists, such as erythromycin can be used, and though it often increases gastric emptying its symptomatic efficacy is limited. Symptomatic relief can be achieved with dopamine 2 receptor antagonists such as metoclopramide, which, however, has little impact on gastric emptying. Gastric pacing by a gastric pacemaker is effective for symptom relief in diabetes mellitus induced gasroparesis, but with only limited efficacy on gastric emptying.

Constipation is a common symptom, and osmotic active preparations (polyethylene glycol) and picosulfate are often effective. Alternating diarrhoea and constipation are often induced by small bowel contamination caused by stagnant content in the small intestine. Small intestine culture or more convenient the hydrogen breath test can disclose the condition, and antibiotics such as tetracycline and/or metronidazole are generally effective, and repeated short courses of treatment can be prescribed when needed. The onset of more continuous diarrhoea is often related to malabsorption, especially of fat and bile acids. Various tests can diagnose the conditions, such as the ^75^Se-homocholicacid-taurine (SeHCAT) test, which diagnose bile acid malabsorption, a condition that often accompany fat malabsorption. Bile acid sequestrates and fat restricted diet, with the help of a dietician to ensure sufficient nutritional intake should be tried. Octreotide, a somatostatin analogue has also been reported to be effective. When treatment fails, and the patient has devastating faecal incontinence, a sigmoid stoma can help the patient to gain control of his/hers bowel movements.

It is important to give the patients adequate supplementation with fat-soluble vitamins and calcium to avoid osteoporosis, and B12 vitamin supplementation may be needed.

Symptoms of adrenal insufficiency can be difficult to distinguish from GI symptoms caused by ATTR amyloidosis. Cortisol supplementation can have a dramatic effect on the patient’s symptoms, including those of orthostatic hypotension.

